# The Effects of Short- and Long-Term Ingestion of Plastic Toxin Bisphenol A on Gastrointestinal Transit Time in Rats

**DOI:** 10.7759/cureus.53694

**Published:** 2024-02-06

**Authors:** Devarshi Dixit, Atanu Roy, Anubhuti Shukla, Parul Sharma, Maloy Mandal

**Affiliations:** 1 Department of Physiology, Institute of Medical Sciences, Banaras Hindu University, Varanasi, IND; 2 Department of Pharmacology, Institute of Medical Sciences, Banaras Hindu University, Varanasi, IND; 3 Department of Physiology, Mata Gujri Memorial (MGM) Medical College, Kishanganj, IND

**Keywords:** endocrine disrupting chemicals (edcs), plastic chemical, charcoal marker method, gastrointestinal transit time, bisphenol a

## Abstract

Introduction

Exposure to bisphenol A (BPA), a toxic chemical released from plastic, affects various body functions, including reproduction, metabolism, and development. The most common route of exposure to BPA is oral, and the gastrointestinal (GI) tract is, therefore, the first body system to be exposed to BPA. BPA has been well-documented to impair gut contractility in rats, in vitro. It may therefore be hypothesized that BPA may adversely affect GI motility and hence slow down the movement of food, resulting in the increased transit of food bolus in the GI tract. There are no reports so far on the effects of BPA on GI transit time.

Objectives

The present study was undertaken to examine the impact of exposure to BPA by a single oral dose (termed as short-term ingestion of BPA) and chronic (28-day) oral dose (termed as long-term ingestion of BPA) on the transit time of food bolus in the gut of adult male albino rats.

Methods and materials

The study was conducted in the Department of Physiology, Institute of Medical Sciences, Banaras Hindu University, Varanasi, Uttar Pradesh, India. In one set of experiments, each animal was fed a food pellet, once (short-term ingestion) containing BPA (2 µg/kg and 50 µg/kg in different groups), and in another set of experiments, each animal was fed a food pellet containing BPA (50 µg/kg/day) for 28 consecutive days (long-term ingestion). Control rats in both sets were fed food pellets without BPA. Subsequently, the gastric transit index (GTI), ileocecal transit index (ICTI), and colonic transit time (CTT) were determined by the standard charcoal marker method.

Results

One-time ingestion of a food pellet containing BPA caused a significant (p < 0.05) drop in the GTI and ICTI and an increase in the CTT with both doses of BPA (2 and 50 µg/kg). Similarly, after chronic (28-day), oral BPA exposure, a significant decrease in the GTI and ICTT and an increase in CTT were observed.

Conclusion

Both short-term (one-time) and long-term (28-day) oral exposure to BPA-containing food harmed GI transit. Slow GI transit may lead to metabolic disorders and GI motility disorders, such as constipation.

## Introduction

The chemical bisphenol A (BPA) is produced in high volume and widely applied in the manufacturing of polycarbonate plastics and epoxy resins. Polycarbonate plastics have extensive usage in making food packaging [1]. Epoxy resins provide an inside coat to certain metallic items, such as food cans, bottle caps, and water supply pipes. BPA-containing plastic also finds wide use in manufacturing baby care items, such as formula food packaging and feeding bottles. Moreover, BPA has applications in making dental implants and sales receipts [2-3].

Food contact plastic has extensive applications in day-to-day life. Easy availability at low cost, strength, and carefree handling make plastic containers suitable for storing, serving, and transporting edibles. There is a constant leaching of BPA from these containers into the edibles, particularly when the containers are recycled, heated, and exposed to non-neutral pH. When BPA-polluted food is consumed, the gastrointestinal (GI) system is the first to be exposed to BPA. BPA is now omnipresent in the environment; hence, exposure of biological systems to BPA has become unavoidable. In several studies, BPA has been found in most human body fluid samples in the American and Asian populations [2-3]. Thus, the people at large are exposed to the risk of the toxic effects of BPA. BPA belongs to a family of endocrine-disrupting chemicals (EDCs) and has estrogenic activity [4]. Evidence of BPA as a potential causative agent for breast, uterine, ovarian, liver, testes, and prostate cancers at toxic doses has been reported [4]. Studies show potential effects on breast oncogenesis at low doses (<25 μg/kg/day) [5]. Moreover, BPA is well-documented to cause developmental defects, reproductive abnormalities, metabolic disorders, neurological abnormalities, immune dysfunction, and prostate cancer [2-3].

The gut is the first organ to be exposed to BPA, and the oral route of exposure is most predominant [3]. A few experimental studies conducted on rats have reported the detrimental impact of BPA on in-vitro gastrointestinal contractility [6-7]. Since BPA has been documented to adversely impact gut contractility in vitro, we hypothesized that oral exposure to BPA may slow down gut motility and delay gastrointestinal transit. However, no report is available regarding any changes in gastrointestinal transit time in response to BPA exposure. Therefore, the present study was undertaken to examine the impact of exposure to BPA by a single oral dose (termed as short-term ingestion of BPA) and chronic (28-day) oral dose (termed as long-term ingestion of BPA) on the transit time of food bolus in the gut of adult male rats.

## Materials and methods

Animals

This study was conducted in the Department of Physiology, Institute of Medical Sciences, Banaras Hindu University, Varanasi, Uttar Pradesh, India. The animal experiments were performed after the approval (Ref. No. Dean/2015/CAEC/1426) by the ethical clearance committee of the institute. Adult male albino rats of Charles Foster strain weighing 150-200 g (three to six months old) were used in the study. The animals were housed in a temperate, humid, and light-controlled room (12:12 h light:dark) with an ad libitum food and water supply.

**Figure 1:**
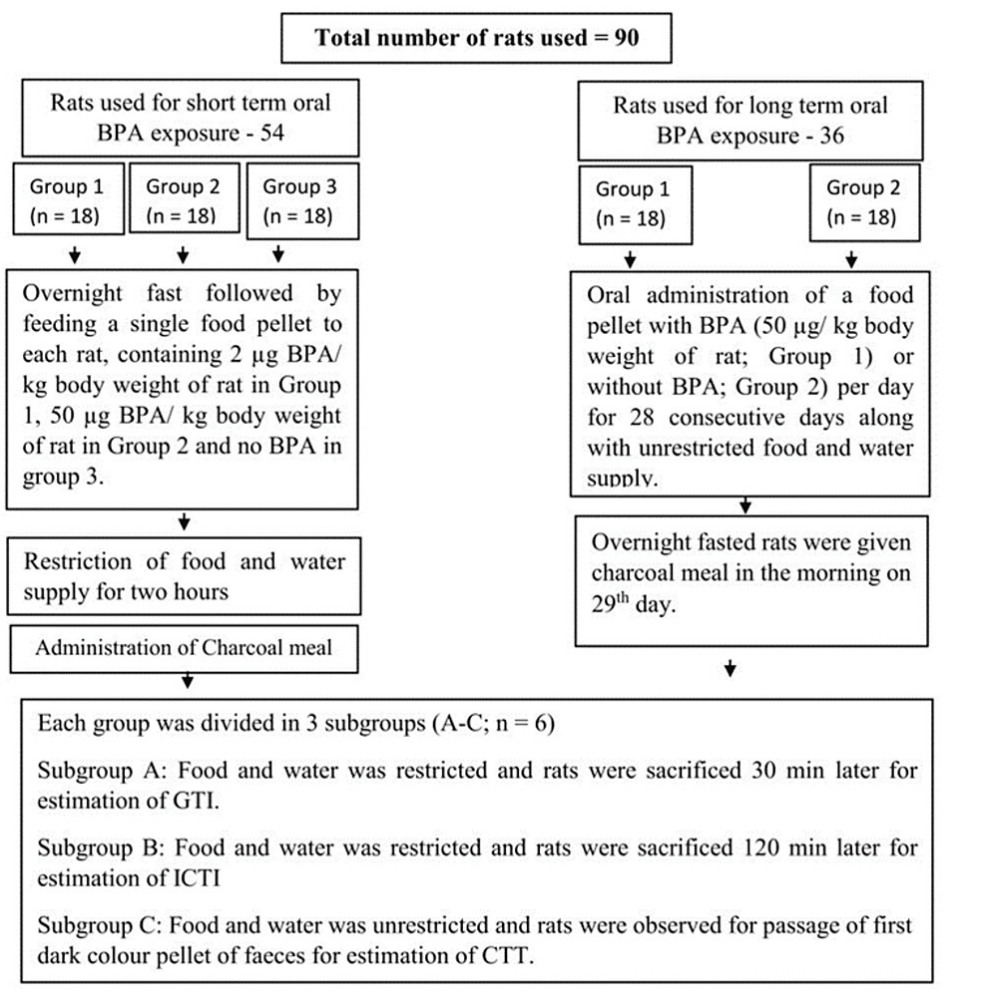
Flow chart showing the number of rats in each group and subgroup and the experimental protocol BPA: bisphenol A; GTI: gastric transit index; ICTI: ileocecal transit index; CTT: colonic transit time; n: number of rats

The flowchart (Figure 1) shows the grouping of animals, the number of animals in each group, and the experimental protocol.

Selection of dose

The selection of the dose of BPA for oral exposure was rationalized based on previous studies [8-10]. Consumption of 50 µg/kg/day of BPA has been considered safe for humans [8] and 2 µg/kg of BPA has been used in earlier studies [9]. Even lower doses of BPA have been shown to produce adverse health effects [10].

BPA was procured from HIMEDIA Laboratories Pvt. Ltd. (Mumbai, India), and activated charcoal was obtained from Qualikems Fine Chemicals Pvt. Ltd. (New Delhi), India.

Short-term ingestion of BPA

Grouping of Animals

A total of 54 randomly selected adult male rats were divided equally into three groups (Groups 1, 2, and 3). After an overnight fast, on the following day, each rat in Group 1 was fed a food pellet containing 2 µg/kg BPA, each rat in Group 2 was fed a food pellet containing 50 µg/kg BPA, and each rat in Group 3 was fed a pellet without BPA. Group 3 served as control.

Preparation of Food Pellet

Each rat was weighed, and the amount of BPA to be mixed in the food pellet was determined according to the rat's weight. BPA was dissolved in olive oil and was mixed with a fixed quantity of rat diet. Two hours after the administration of the food pellet, the marker (charcoal meal) was given. During these two hours, no food or water was provided to the animals.

Estimation of the Gastric Transit Index (GTI), Ileocecal Transit Index (ICTI), and Colonic Transit Time (CTT)

 Each of the three groups (1-3) was divided into three subgroups (A-C), with six rats in each subgroup. After administration of charcoal meal, no food and water were provided to subgroups A and B of each group. Subgroups A and B were later subjected to the recording of GTI and ICTI, respectively. On the other hand, ad libitum food supply was maintained for subgroup C. The subgroup C was later subjected to the recording of CTT.

Long-term ingestion of BPA

Grouping of Animals

A total of 36 rats were recruited in this part of the study and were divided randomly and equally into two groups (Group 1 and Group 2). In Group 1, the rats were fed one food pellet containing BPA (50 µg/kg body wt/day) for consecutive 28 days. After consuming the BPA pellet, rats had free access to the regular food pellets (without BPA) for the rest of the day. Similarly, tap water supply was ad libitum. In Group 2, rats were fed a similar food pellet without BPA for 28 days without any restrictions on food and water supply. On the 29th day, all the rats were overnight fasted.

Estimation of the GTI, ICTI, and CTT

The rats in both groups (Group 1 and Group 2) were randomly and equally divided into three subgroups (A, B, and C) to assess the GTI, ICTI, and CTT, respectively, using the marker (charcoal meal). As described earlier, after administration of the charcoal meal, no food and water were provided to subgroups A and B of each group (1-2). Subgroups A and B were later subjected to the recording of GTI and ICTI, respectively. On the other hand, ad libitum food supply was maintained for subgroup C of each group (1-2). Subgroup C was later subjected to the recording of the CTT.

Dissection and procedure for recording of GTI, ICTI, and CTT

**Figure 2:**
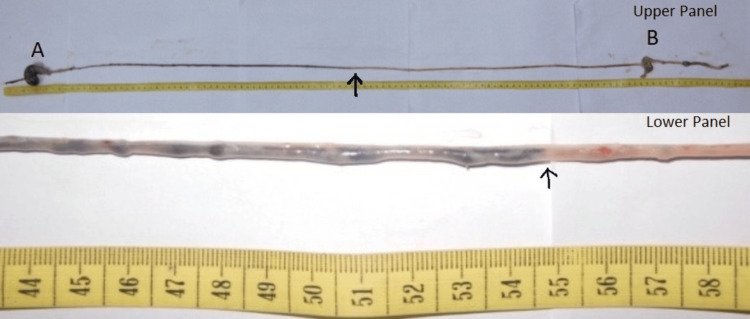
Upper panel: A sample of the entire length of gut removed from the rat after 30 minutes of charcoal meal ingestion. Lower panel: The same with an enlarged view. Please note the dark-colored food passing along the length of the small intestine. The black arrow indicates the point of the gut to which the dark marker (charcoal mixed food) traversed. This length of the rat gut up to this point was noted for the calculation of GTI. Also, note the two dilated segments indicating stomach (A) and cecum (B). GTI: gastric transit index

**Figure 3:**
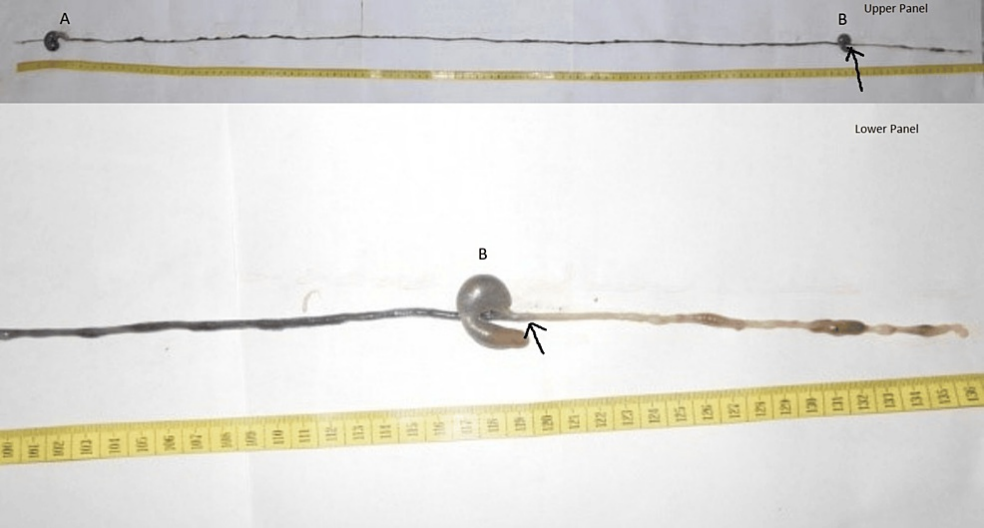
Upper panel: A sample of gut dissected out after 120 min of charcoal meal ingestion for recording the ICTI. Lower panel: The same with an enlarged view. Please note the dark-colored food crossed the ileoceal junction. The black arrow indicates the point of the gut to which the dark marker (charcoal mixed food) traversed. This length of the rat gut up to this point was noted for the calculation of ICTI. Also, note the two dilated segments indicating stomach (A) and cecum (B). ICTI: ileocecal transit index

The GTI and ICTI were determined after the minor modification of the procedure described earlier [11-12]. Thirty minutes and 120 minutes post ingestion of the charcoal meal (marker), the rats were sacrificed for the assessment of the GTI and ICTI, respectively. The method used for the sacrifice was cervical dislocation. The abdomen was opened immediately by a midline incision, and the entire gut length from the esophagus to the anus was dissected and measured. The point of the intestine at which the marker reached was noted (Figure 2 and Figure 3). The total length of the intestine was measured, and the length of the intestine through which the marker traveled was expressed as a percentage (%) of the total intestinal length [11].

**Figure 4:**
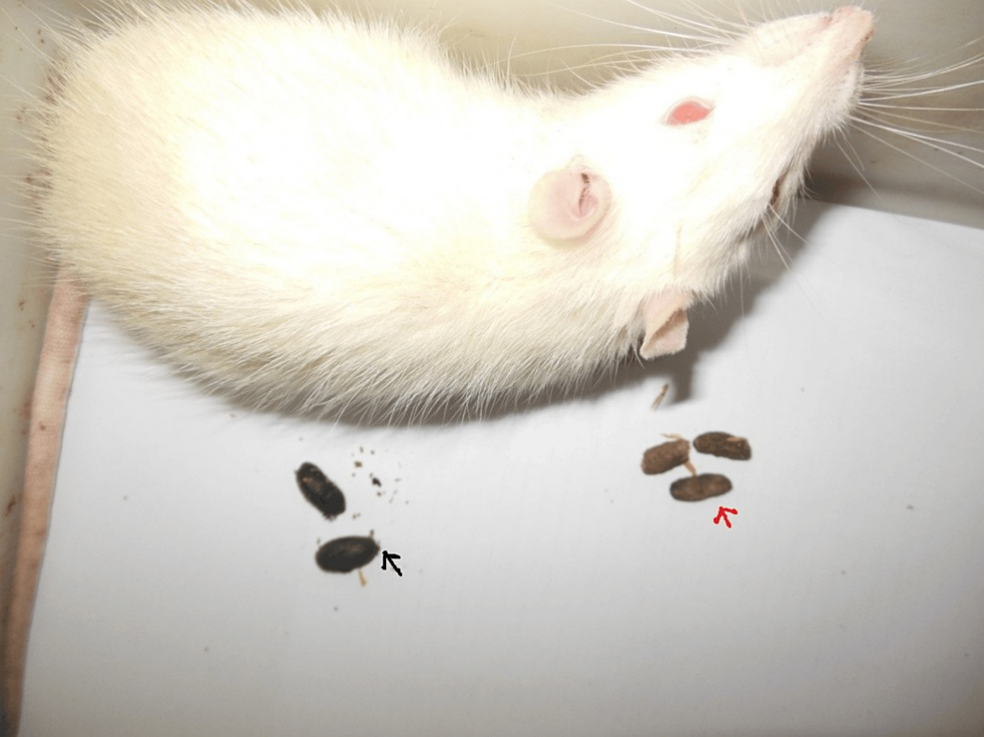
Image showing charcoal stained (dark) and normal fecal pellets indicated by black and red arrows, respectively. The CTT was determined based on the time taken for the elimination of the first dark pellet after a charcoal meal. CTT: colonic transit time

For the recording of the CTT, the rats were left under continuous observation after giving a charcoal meal, and time was noted when the first dark-colored pellet of feces was expelled (Figure 4). The dark color of the pellet was due to charcoal meal. CTT was expressed in terms of time (minutes) [13].

Data analysis and statistics

The data were expressed as mean ± SD values, and different groups were compared by the one-way analysis of variance (ANOVA) test, post-hoc Tukey test, and independent t-test. P value < 0.05 was considered significant.

## Results

Effects of short-term ingestion of BPA on GI transit time

Effects of Short-Term Ingestion of BPA on the GTI

**Figure 5:**
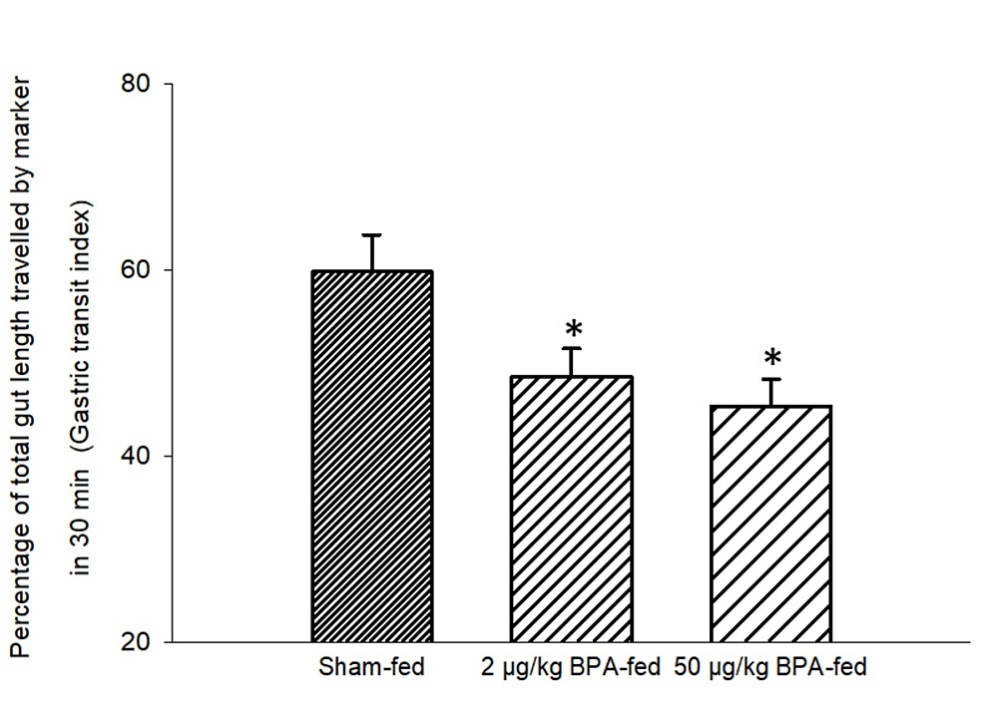
Bar diagram showing the mean ± SD values of the percentage of the total gut length traveled by the marker in 30 minutes after the ingestion of charcoal meal (gastric transit index, GTI) in different groups. Group 1 (n = 6) and Group 2 (n = 6) were exposed to a single food pellet containing 2 µg/kg BPA and 50 µg/kg BPA, respectively, while Group 3 (n = 6) was exposed to a single food pellet without BPA (sham-fed). The GTI in both BPA-fed groups was significantly different from that of the sham–fed group. The asterisks indicate a significant difference (p < 0.05) from the sham-fed group (one-way ANOVA test; post-hoc Tukey test). BPA: bisphenol A; GTI: gastric transit index; ANOVA: analysis of variance; SD: standard deviation, n: number of animals in each group

Thirty minutes after the ingestion of charcoal meal, the marker reached 48.46 ± 3.06% of the total gut length in Group 1 (2 µg/kg BPA-fed group), 45.29 ± 2.92% of the total gut length in Group 2 (50 µg/kg BPA-fed group), and 59.77 ± 3.98% of the total gut length in Group 3 (control animals) (Figure 5). Thus, there was an 11.31% decrease in the GTI for the rats treated with 2 µg/kg BPA and a 14.48% decrease in the GTI for the rats treated with 50 µg/kg BPA as compared to the sham-fed rats. Therefore, there was a significant alteration in the GTI in both the BPA-fed groups as compared to the control group (p < 0.05, one-way ANOVA test; post-hoc Tukey test).

Effects of Short-Term Ingestion of BPA on the ICTI

**Figure 6:**
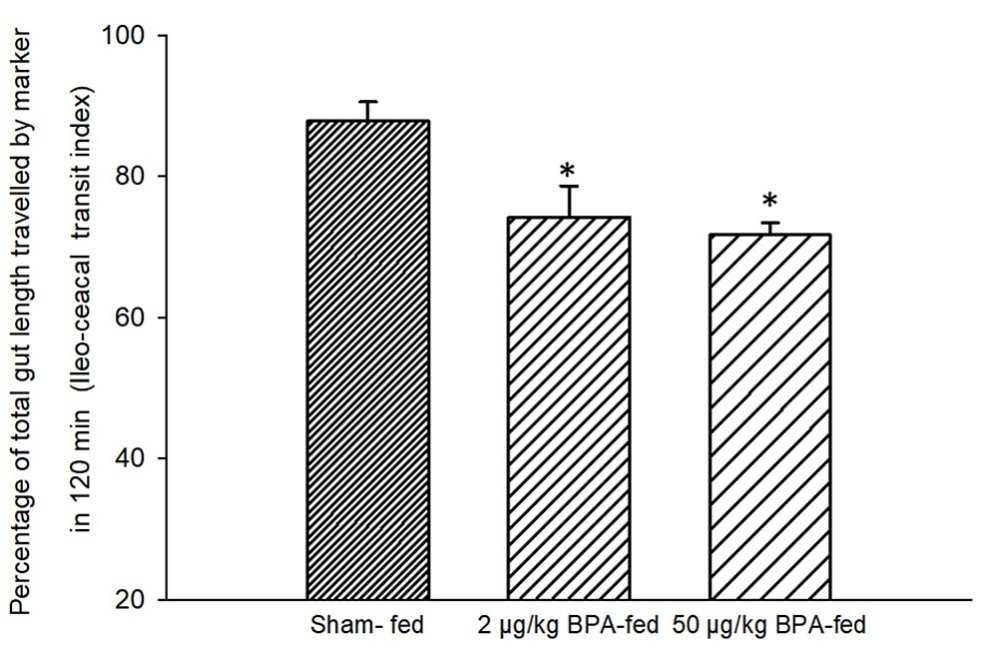
Bar diagram showing the mean ± SD values for the percentage of the total gut length traveled by the marker in 120 minutes after the ingestion of the charcoal meal (ileocecal transit index, ICTI) in different groups. Group 1 (n = 6) and Group 2 (n = 6) were exposed to a single food pellet containing 2 µg/kg BPA and 50 µg/kg BPA, respectively, while Group 3 (n = 6) was exposed to a single food pellet without BPA (sham-fed). The ICTI in both BPA-fed groups was significantly different from the sham–fed group. The asterisks indicate a significant difference (p < 0.05) from the sham-fed group (one-way ANOVA test; post-hoc Tukey test). BPA: bisphenol A; ICTI: ileocaecal transit index; ANOVA: analysis of variance; SD: standard deviation; n: number of animals in each group

One hundred twenty minutes after the ingestion of the charcoal meal, the marker reached 74.21 ± 4.38% and 71.69 ± 1.70% of the total gut length in the 2 µg/kg BPA-fed rats (Group 1) and 50 µg/kg BPA-fed rats (Group 2), respectively (Figure 6) as compared to the sham-fed group (Group 3; control rats), which showed an average of 87.86 ± 2.70% of the total gut length traversed by the marker (Figure 6). Thus, there was a small but statistically significant decrease (13.65% and 16.17%) in the ICTI for the 2 µg/kg and 50 µg/kg BPA-fed group of rats, respectively (p < 0.05, one-way ANOVA test; post-hoc Tukey test). 

Effects of Short-term Ingestion of BPA on the CTT

The CTT in the 2 µg/kg BPA-fed rats (Group 1), 50 µg/kg BPA-fed rats (Group 2), and sham-fed rats (Group 1) was recorded as 405.00 ± 22.14 minutes, 415.00 ± 29.66 minutes, and 365.00 ± 24.70 minutes, respectively (Table 1). The increase in the CTT for both BPA-fed groups as compared to the sham-fed group of rats was statistically significant (p < 0.05, one-way ANOVA test; post-hoc Tukey test).

**Table 1:** Time taken for the excretion of the first marker pellet (colonic transit time, CTT) after exposure to BPA by a single oral dose in different groups. Group 1 (n = 6) and Group 2 (n = 6) were exposed to a single food pellet containing 2 µg/kg BPA and 50 µg/kg BPA, respectively, while Group 3 (n = 6) was exposed to a single food pellet without BPA (sham-fed). The mean ± SD values are in minutes. The CTT in both BPA-fed groups was significantly different from that of the sham–fed group. The asterisks indicate a significant difference (p < 0.05) from the sham-fed group (one-way ANOVA test; post-hoc Tukey test). CTT: colonic transit time; BPA: bisphenol A; ANOVA: analysis of variance; SD: standard deviation; n: number of animals in each group

Groups	Treatment	Mean CTT (minutes)	SD	p-values
Group 1 (n = 6)	2 µg/kg BPA-fed	405.00*	22.14	p = 0.041*
Group 2 (n = 6)	50 µg/kg BPA-fed	415.00*	29.66	p = 0.011*
Group 3 (n = 6)	Sham-fed	365.00	24.70	-

Effects of long-term ingestion of BPA on the GTI, ICTI, and CTT

**Figure 7:**
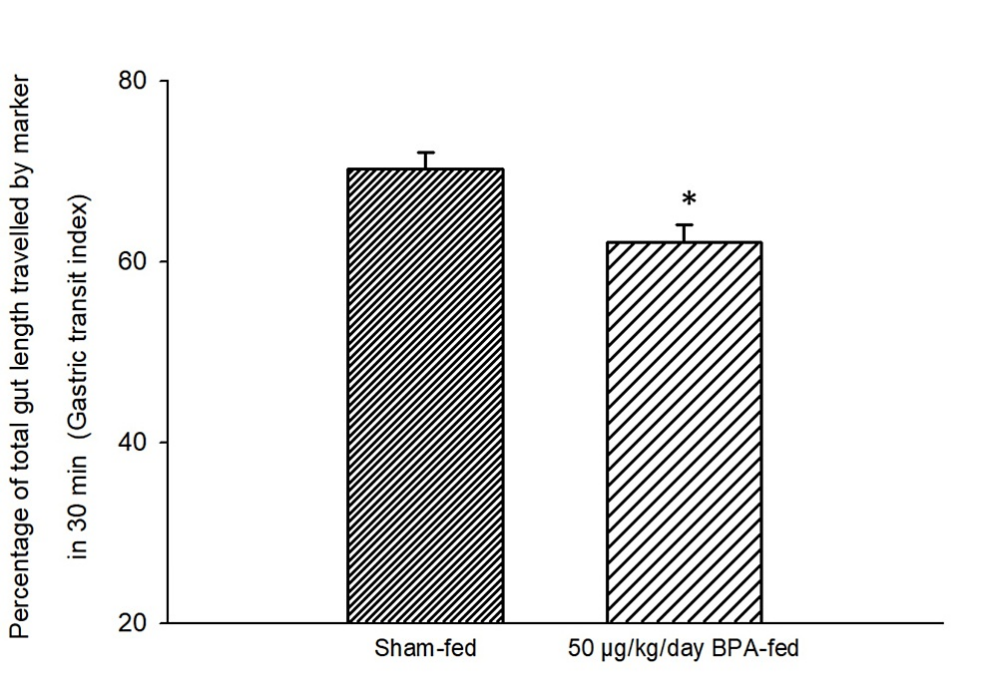
Bar diagram showing the mean ± SD values of the percentage of the total gut length traveled by the marker in 30 minutes after the ingestion of the charcoal meal (gastric transit index, GTI) in different groups. Group 1 (n = 6) was exposed to a food pellet containing 50 µg/kg/day BPA while Group 2 (n = 6) was exposed to a food pellet without BPA (sham-fed) for 28 consecutive days. The GTI in the BPA-fed group was significantly different from that of the sham–fed group. The asterisk indicates a significant difference (p < 0.05) from the sham–fed group (independent t-test). GTI: gastric transit index; BPA: bisphenol A; SD: standard deviation; n: number of animals in each group

In the BPA-fed group (Group 1), the marker reached 62.20 ± 1.91% of the total gut length 30 minutes after the ingestion of the charcoal meal. In control animals (Group 2), the marker reached an average of 70.24 ± 1.88% of the total gut length 30 minutes after the ingestion of the charcoal meal (Figure 7). Thus, there was about an 8% decrease in the GTI in the BPA-fed group. This alteration in the GTI in the BPA-fed group as compared to the sham-fed group was found to be statistically significant (p < 0.001; independent t-test).

**Figure 8:**
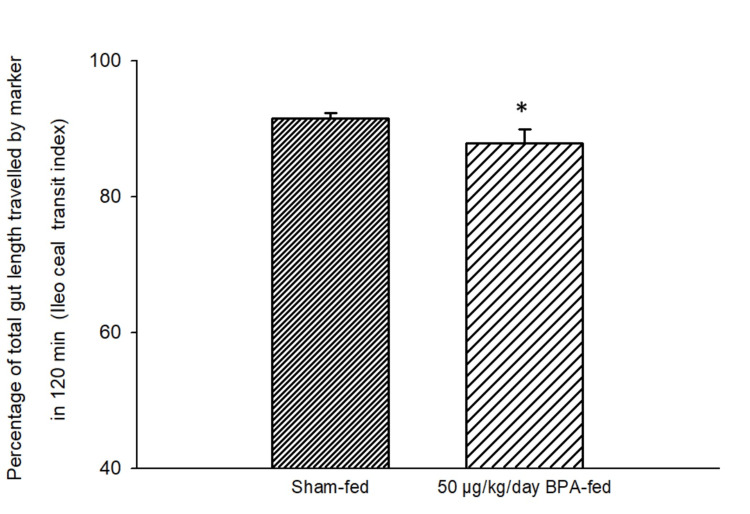
Bar diagram showing the mean ± SD values of the percentage of the total gut length traveled by the marker in 120 minutes after the ingestion of the charcoal meal (ileoceacal transit index, ICTI) in different groups. Group 1 (n = 6) was exposed to a food pellet containing 50 µg/kg/day BPA while Group 2 (n = 6) was exposed to a food pellet without BPA (sham-fed) for 28 consecutive days. The ICTI in the BPA-fed group was significantly different from the sham–fed group. The asterisk indicates a significant difference (p < 0.05) as compared to the sham-fed group (independent t-test). ICTI: ileoceacal transit index; BPA: bisphenol A; SD: standard deviation; n: number of animals in each group

Similarly, there was a significant (p < 0.05, independent t-test) change in the ICTI (Figure 8) after 28 days of BPA (50 µg/kg/day) feeding as compared to the sham-fed group.

Table 2 shows that there was a significant (p < 0.05, independent t-test) change in the CTT after 28 days of BPA (50 µg/kg/day) feeding as compared to the sham-fed group.

**Table 2:** Time taken for the excretion of the first marker pellet (colonic transit time, CTT) after chronic (28-day) oral exposure to BPA. Group 1 (n = 6) was exposed to a food pellet containing 50 µg/kg/day BPA while Group 2 (n = 6) was exposed to a food pellet without BPA (sham-fed) for 28 consecutive days. The mean ± SD values are in minutes. The CTT in the BPA-fed group was significantly different from that of the sham–fed group. The asterisk indicates a significant difference (p < 0.05) from the sham-fed group (independent t-test). CTT: colonic transit time; BPA: bisphenol A; ANOVA: analysis of variance; SD: standard deviation; n: number of animals in each group

Groups	Treatment	Mean	SD	p-value
Group 1 (n = 6)	50 µg/kg/day BPA-fed	484.17	39.80	0.034*
Group 2 (n = 6)	Sham-fed	530.83	24.38	

## Discussion

This is a preliminary study showing the impact of BPA ingestion on the transit time of food in the GI tract. The GTI, ICTI, and CTT were recorded following the short-term and long-term ingestion of BPA. Rats fed with a single food pellet containing BPA (2 µg BPA/kg and 50 µg BPA/kg in different groups) showed a decreased GTI, decreased ICTI, and increased CTT significantly (Figures 5-6, Table 1) as compared to the control (sham-fed rats). A decreased GTI signifies an increased transit time and therefore indicates a slower movement of food bolus containing BPA in the treated rats as compared to the control (sham-fed) rats. Movement of food having a higher content of BPA (50 µg/kg) was even slower than that of food containing a lower amount of BPA (2 µg/kg). These results signified the role of BPA in reducing gut motility. Similarly, after chronic (28-day) oral exposure to BPA, the GTI and ICTI were found to be significantly less and the CTT was significantly more in the BPA-treated rats as compared to the control rats.

The mechanism by which the BPA reduces GI motility is unclear from the present experiments. BPA is a known endocrine disruptor with estrogen-like actions [2]. Estrogen has been reported to delay gastric emptying time or decrease the GTI [14]. Thus, the estrogen-like action of BPA may be responsible for delaying gastric emptying time or reducing GI transit in the present investigation. Another possible factor for the relaxation of the intestinal smooth muscle, which inhibits GI motility is nitric oxide (NO). NO is a ubiquitous transmitter located in all tissues [15]. Endothelial cells possess the synthetic enzyme eNOS (endothelial nitric oxide synthase), which generates NO from arginine and releases NO [15]. The NO is a potent smooth muscle relaxant and relaxes all the smooth muscles in the body [6,16-17]. There is ample evidence to suggest the involvement of nitric oxide in regulating intestinal contractility [6,18-19]. A recent study claimed that BPA caused a significant inhibition of duodenal movement by involving NO [6]. The same effect was reflected in the present study in the ICTI and CTT, suggesting the possibility of BPA-induced NO mechanisms in the colon and rectum. In addition to these mechanisms, one may argue that the olive oil used to dissolve BPA could also contribute to the delayed transit of food bolus [20]. However, it is unlikely in the present investigation because the control rats also received the same olive oil in the same quantity.

Limitations

Although it is a preliminary study, findings must be confirmed using more samples and radiological techniques. A further evaluation with a higher dose and extended feeding period may also be required.

BPA is an endocrine-disrupting chemical with estrogenic properties. Gender may influence the effect of BPA on the transit time, which may be investigated by using both male and female rats in different groups. In the present study, only male rats were used to explore the effect of BPA on the GI transit time, and any possible influence of gender on the effect of BPA on the transit time was not explored. Moreover, the estimation of serum levels of BPA in treated and control rats and the histological examination of gut tissue in chronically BPA-exposed rats were not performed.

## Conclusions

Thus, from the present study, it may be concluded that short- and long-term ingestion of BPA-containing food pellets delayed GI transit time by mechanisms yet to be delineated.

The impact of delayed transit time may be clinically relevant since BPA has been implicated in the development of metabolic disorders. Another possible effect of delayed transit time may be gut motility disorders, such as constipation. This may be avoided by minimizing the usage of food-contact plastic in day-to-day life.

## References

[1] Maršálek P, Kovaříková S, Lueerssen F, Večerek V (2022). Determination of bisphenol A in commercial cat food marketed in the Czech Republic. J Feline Med Surg.

[2] Sharma P, Mandal MB, Katiyar R, Singh SP, Birla H (2021). A comparative study of effects of 28-day exposure of bisphenol A and bisphenol S on body weight changes, organ histology, and relative organ weight. Int J Appl Basic Med Res.

[3] Sharma P, Bhagat P, Mandal MB, Singh TB (2021). Assessment of awareness regarding health hazards of plastic chemicals and their warning label among a sample population of Varanasi city: a cross-sectional study. J Pharm Bioallied Sci.

[4] Wazir U, Mokbel K (2019). Bisphenol A: a concise review of literature and a discussion of health and regulatory implications. In Vivo.

[5] Wang Z, Liu H, Liu S (2017). Low-dose bisphenol A exposure: a seemingly instigating carcinogenic effect on breast cancer. Adv Sci (Weinh).

[6] Sarkar K, Tarafder P, Paul G (2016). Bisphenol A inhibits duodenal movement ex vivo of rat through nitric oxide-mediated soluble guanylyl cyclase and α-adrenergic signaling pathways. J Appl Toxicol.

[7] Nirja K, Sharma P, Tiwari AK, Mandal MB (2018). Plastic toxin bisphenol-A depresses the contractile activity of rat ileum and colon in vitro. Indian J Physiol Pharmacol.

[8] Calafat AM, Ye X, Wong LY, Reidy JA, Needham LL (2008). Exposure of the U.S. population to bisphenol A and 4-tertiary-octylphenol: 2003-2004. Environ Health Perspect.

[9] Nagel SC, vom Saal FS, Thayer KA, Dhar MG, Boechler M, Welshons WV (1997). Relative binding affinity-serum modified access (RBA-SMA) assay predicts the relative in vivo bioactivity of the xenoestrogens bisphenol A and octylphenol. Environ Health Perspect.

[10] Markey CM, Wadia PR, Rubin BS, Sonnenschein C, Soto AM (2005). Long-term effects of fetal exposure to low doses of the xenoestrogen bisphenol-A in the female mouse genital tract. Biol Reprod.

[11] de-Oliveira GR, Gondim FA, da-Graça JR (1998). Acute blood volume expansion delays the gastrointestinal transit of a charcoal meal in awake rats. Braz J Med Biol Res.

[12] Datta U (2001). Effect of heat stress on gastro-intestinal motility in young albino rats. Indian J Physiol Pharmacol.

[13] Tuleu C, Andrieux C, Boy P, Chaumeil JC (1999). Gastrointestinal transit of pellets in rats: effect of size and density. Int J Pharm.

[14] Heinemann A, Pieber D, Holzer P (2002). Inhibition by female sex steroids of peristalsis in the guinea pig small intestine. Digestion.

[15] Lundberg JO, Weitzberg E (2022). Nitric oxide signaling in health and disease. Cell.

[16] Gupta H, Deshpande SB (2018). Bisphenol A decreases the spontaneous contractions of rat uterus in vitro through a nitrergic mechanism. J Basic Clin Physiol Pharmacol.

[17] Li H, Xia N, Hasselwander S, Daiber A (2019). Resveratrol and vascular function. Int J Mol Sci.

[18] Yuan F, Ren H, Tan W, Wang Y, Luo H (2022). Effect of phosphodiesterase-4 inhibitor rolipram on colonic hypermotility in water avoidance stress rat model. Neurogastroenterol Motil.

[19] Yip JL, Balasuriya GK, Spencer SJ, Hill-Yardin EL (2022). Examining enteric nervous system function in rat and mouse: an interspecies comparison of colonic motility. Am J Physiol Gastrointest Liver Physiol.

[20] Gentilcore D, Chaikomin R, Jones KL (2006). Effects of fat on gastric emptying of and the glycemic, insulin, and incretin responses to a carbohydrate meal in type 2 diabetes. J Clin Endocrinol Metab.

